# Virtual auscultation course for medical students via video chat in times of COVID-19

**DOI:** 10.3205/zma001395

**Published:** 2020-12-03

**Authors:** Nils Rüllmann, Unaa Lee, Kathrin Klein, Bastian Malzkorn, Ertan Mayatepek, Matthias Schneider, Carsten Döing

**Affiliations:** 1 Heinrich Heine University Düsseldorf, Medical Faculty, Office of the dean of studies, Düsseldorf, Germany; 2 University Children’s Hospital Düsseldorf, Department of General Pediatrics, Neonatology and Pediatric Cardiology, Düsseldorf, Germany; 3 Heinrich Heine University Düsseldorf, Medical Faculty, Düsseldorf, Germany; 4 University Hospital Düsseldorf, Division of Cardiology, Pneumology and Angiology, Düsseldorf, Germany

**Keywords:** COVID-19, distance learning, virtual auscultation, case-based learning, peer-teaching, skills lab, video chat

## Abstract

**Introduction: **Auscultation skills are among the basic techniques to be learned in medical school. Such skills are achieved through supervised examination of patients often supported by simulator-based learning.

The emergence of COVID-19 has disrupted and continues to hinder hands-on on-site medical training on a global scale.

**Project description: **An effective virtual auscultation course was established in times of contact restrictions due to COVID-19 at the Medical Faculty of the Heinrich Heine University Düsseldorf. The interactive case-based webinar was designed to improve listening techniques, description and interpretation of auscultation findings in an off-site context. Clinical cases with pre-recorded auscultation sounds and additional case-based diagnostics were presented. The course focused on common heart murmurs including aortic and mitral valve stenosis and regurgitation as well as congenital heart defects (ventricular septal defect and patent ductus arteriosus).

**Results: **The course was well received by the students and assessed as being useful and instructive. Assessment of learning effects, such as detection of pathological findings before and after training, is ongoing as part of a subsequent trial.

**Conclusion:** Virtual interactive learning using a sound simulation lesson with clinical case presentations via video chat can well be used as a supplement to practical auscultation training. This learning format could also play a useful role in the curriculum of medical studies once contact restrictions are revoked.

## Zusammenfassung

**Einleitung: **Auskultationsfertigkeiten gehören zu den grundlegenden Fertigkeiten, die in der medizinischen Ausbildung erlernt werden müssen. Diese werden durch supervidierte Patientenuntersuchung erworben und können durch simulatorbasiertes Lernen unterstützt werden.

Die COVID-19-Pandemie hat die praktisch medizinische Ausbildung vor Ort weltweit beeinträchtigt und behindert diese weiterhin.

**Projektbeschreibung: **An der Medizinischen Fakultät der Heinrich-Heine-Universität Düsseldorf wurde in Zeiten von Kontaktbeschränkungen aufgrund von COVID-19 ein virtueller Auskultationskurs etabliert. Das interaktive, fallbasierte Online-Seminar wurde entwickelt, um die Hörtechniken, die Beschreibung und die Interpretation von Auskultationsbefunden in einem Off-Site-Kontext zu verbessern. Es wurden klinische Fälle mit voraufgezeichneten Auskultationsbefunden und zusätzlicher fallbasierter Diagnostik vorgestellt. Der Kurs konzentrierte sich auf häufige Herzgeräusche einschließlich der Stenosen und Insuffizienzen von Aorten- und Mitralklappe sowie auf angeborene Herzfehler (Ventrikelseptumdefekt und persistierender Ductus arteriosus).

**Ergebnisse: **Der Kurs wurde von den Studierenden gut angenommen und als nützlich und lehrreich bewertet. Die Beurteilung von Lerneffekten, wie z.B. das Erkennen von pathologischen Befunden vor und nach dem Training, wird im Rahmen einer Folgestudie untersucht.

**Schlussfolgerung: **Virtuelles interaktives Lernen mittels Geräuschsimulationseinheiten mit klinischen Fallpräsentationen via Videokonferenz kann als Ergänzung zum praktischen Auskultationstraining eingesetzt werden. Dieses Lernformat könnte auch weiterhin im Curriculum des Medizinstudiums genutzt werden, sobald die Kontaktbeschränkungen aufgehoben werden.

## Introduction

Auscultation skills are among the most important basic examination techniques to be learned in medical school [1]. The mastery of cardiac, pulmonary and abdominal auscultation requires the development of complex acoustic skills. These are taught in examination training courses, at the bedside and in theoretical lectures. During internships or clinical rotations, the quality of auscultation training varies widely [2], [3]. Bedside teaching is limited by a high student-to-patient ratio and the variability of clinical presentations [4]. Examining uncooperative patients (e.g. children), lack of equipment for simultaneous auscultation, and infrequent exposure to rare physical findings are other difficulties [4], [5].

Novel methods to teach auscultation include use of patient simulators, multimedia teaching programs or the use of electronic stethoscopes [5], [6], [7], [8], [9], [10].

During the COVID-19 pandemic, medical students were temporarily unable to attend classes and contact restrictions obstructed interaction with patients. Therefore, there was an urgent need for alternative training methods [11]. Pre-recorded auscultation sounds have been used for teaching in medical school and in continuing clinical education for many years [12], [13], [14].

A virtual auscultation course was introduced using clinical cases and audio auscultation files to mitigate the lack of on-site practical training. Video chat as a synchronous communication format was chosen as it most closely resembles the interactive character of face-to-face teaching.

## Project description

**Figure 1:**
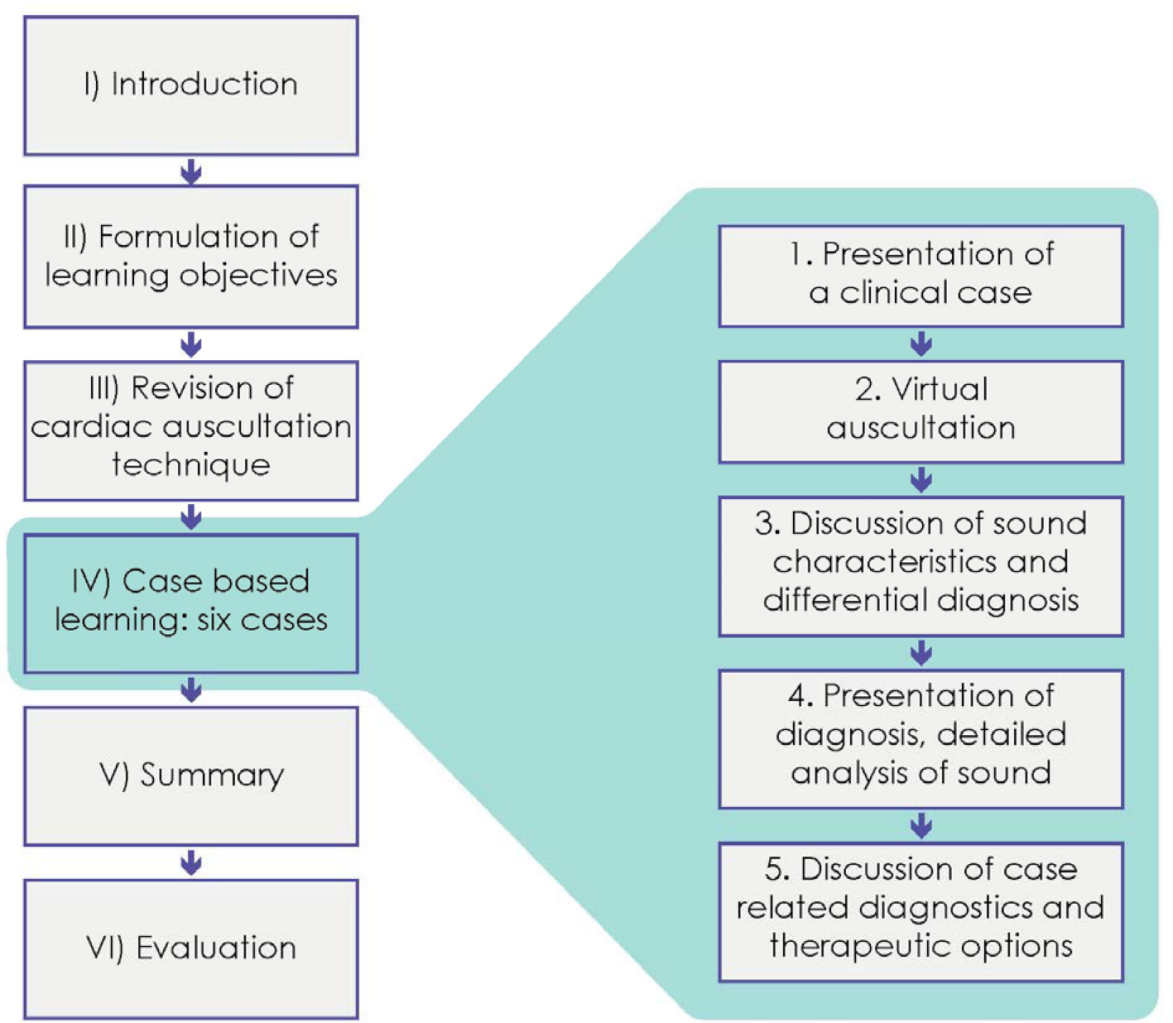
Structure of the virtual auscultation course

An interactive case-based online course was designed at short notice to improve listening techniques, description and interpretation of auscultation findings in an off-site context. Figure 1 (Fig. 1) shows the structure of the course.

Clinical cases with pre-recorded auscultation sounds and additional case-based diagnostics were presented, adapted to the model of case-based-learning [15]. The clinical knowledge was deepened by discussing necessary diagnostics, differential diagnoses and therapy. The peer-teaching course was designed by a medical student and supervised by consultant pediatric and adult cardiologists.

The virtual auscultation course focused on common heart murmurs such as aortic and mitral valve stenosis and regurgitation as well as congenital heart defects (ventricular septal defect and patent ductus arteriosus). Audio auscultation files generated with an analogue simulator for heart sounds and murmurs were used. The files are part of the online learning program Clinisurf [https://clinisurf.elearning.aum.iml.unibe.ch/] at the University of Bern, Switzerland, and used with kind permission.

The course took place via video chat on Microsoft (MS) Teams [https://www.microsoft.com/de-de/]. Slides created with MS PowerPoint were uploaded and shared with participants. The auscultation sounds were embedded into the slides as audio files. Slides uploaded in MS Teams allowed participants to play auscultation sounds individually.

The course started with a recapitulation of stethoscope placement and heart sounds followed by instructions on describing auscultation findings.

Heart murmurs were presented in the context of patient cases: Students were introduced to patients who they virtually auscultated via headphones. After description of the sound’s character and discussion of differential diagnoses, the correct diagnosis was presented.

Sounds and murmurs were visualized using diagrams and compared with other auscultation findings. Videos, used with friendly permission of AMBOSS [https://www.amboss.com/de], were shown to visualize auscultation positions. Finally, diagnostic and therapeutic options were discussed.

The course lasted two hours with 6 to 7 participants. Participants were final year students in practical training and undergraduate students who completed their internship preparatory course. The course was offered weekly in after-work hours, thus allowing practical year students to participate. The course was evaluated by participants using the Medical Faculty's online evaluation form as routinely used for curricular courses.

## Results

All courses offered were fully booked, indicating a high demand for elective virtual courses as an alternative to purely theoretical learning, especially during COVID-19-related contact restrictions. The course was held 13 times in the summer term (total participants n=72).

The participants were asked to evaluate the course using a six-level Likert scale (best score=1) and free text comments. The media used was considered appropriate (MV=1,2, SD=0,5, n=63). Participants assessed their recognition of auscultation findings after completion of the course as good (MV=1,7; SD=0,7, n=63) and indicated a high level of satisfaction (MV=1.2; SD=0.5, n=64).

In comments, participants recommended that the course could be established in the regular curriculum and reported that in courses with bedside teaching there is not always enough time for everyone to individually auscultate patients. Critical feedback referred to sporadic technical problems with image and sound quality due to connectivitiy issues.

## Discussion

Due to the voluntary nature of participation and the presumably high motivation of participants, these results are only first trends.

Time constraints during the initial pandemic prohibited a formal assessment of the effectiveness and sustainability of the virtual course format. The positive reception justifies the continuation in the upcoming term with initiation of a randomised controlled prospective study of teaching effectivity.

Strengths of the virtual teaching concept are the possible scalability, the independence of location and time and the possible curricular anchoring in preparation for cardiological bedside teaching in the sense of blended learning. The project is transferable in terms of content as well as conceptually and technically.

Limitations are technical requirements, missing patient contact as well as the missing use of the own stethoscope with identification of the pattern of heart murmur radiation across the thorax.

## Conclusion

The course was met with a high level of demand, acceptance and satisfaction demonstrating that virtual courses can be an alternative for small group lessons in the absence of classroom teaching.

Student interest in the courses underscores the importance of clinical training to improve auscultation skills. Following the concept of blended learning, the range of courses will include hands-on courses using auscultation simulators as soon as on-site teaching is reinstated. This combined approach can lead to an additional benefit and further acquisition of clinical skills.

## Competing interests

The authors declare that they have no competing interests.
